# Efficient generation of human immune system rats using human CD34^+^ cells

**DOI:** 10.1016/j.stemcr.2024.07.005

**Published:** 2024-08-15

**Authors:** Séverine Ménoret, Florence Renart-Depontieu, Gaelle Martin, Kader Thiam, Ignacio Anegon

**Affiliations:** 1 Nantes Université, CHU Nantes, Inserm, CNRS, SFR Santé, Inserm UMS 016, CNRS UMS 3556, F-44000 Nantes, France; 2 INSERM, Centre de Recherche en Transplantation et Immunologie UMR1064, Nantes Université, Nantes, France; 3 genOway, 69007 Lyon, France

**Keywords:** immunodeficient rodents, human immune system mice, regenerative medicine, cancer models, transplantation, immunotherapy

## Abstract

Human immune system (HIS) mice generated using human CD34^+^ hematopoietic stem cells serve as a pivotal model for the *in vivo* evaluation of immunotherapies for humans. Yet, HIS mice possess certain limitations. Rats, due to their size and comprehensive immune system, hold promise for translational experiments. Here, we describe an efficacious method for long-term immune humanization, through intrahepatic injection of hCD34^+^ cells in newborn immunodeficient rats expressing human SIRPα. In contrast to HIS mice and similar to humans, HIS rats showed in blood a predominance of T cells, followed by B cells. Immune humanization was also high in central and secondary lymphoid organs. HIS rats treated with the anti-human CD3 antibody were depleted of human T cells, and human cytokines were detected in sera. We describe for the first time a method to efficiently generate HIS rats. HIS rats have the potential to be a useful model for translational immunology.

## Highlights

• Immunodeficient/hSIRPα^+^ rats were efficiently immune humanized by hCD34^+^ cells
• hCD45^+^ cells in the blood of rats were equivalent to immune humanized mice
• Contrary to mice, the proportion of T and B cells in rats was as in human blood
• Thymus, bone marrow, spleen, and lymph nodes of rats were also immune humanized

## Teaser

In this article, Anegon et al. show an efficient method to generate human immune system (HIS) rats using hCD34^+^ cells. Numbers of hCD45^+^ cells in HIS rats’ blood were equivalent to HIS mice, but contrary to mice, the proportion of T and B cells was as in human blood. HIS rats showed high percentages of hCD45^+^ cells in lymphoid organs.

## Introduction

Mouse immunodeficient models have proven instrumental in grafting human cells and tissues, facilitating the analysis of their *in vivo* functions under both healthy and diseased conditions (Adigbli et al., 2020; Shultz et al., 2007). Among these tissues, human immune system (HIS) mice have paved the way for significant translational advancements in the manipulation of human immune responses *in vivo* (Adigbli et al., 2020).

Immunodeficient animals of other species generated through targeting of key immune genes have certain specific advantages over mice (Adigbli et al., 2020). Rats present several advantages for immune humanization, including being a small animal model as mice, but they have a normal complement system, whereas NOD-derived mouse strains have an absent complement cascade. Most other mouse strains (including C57BL/6 and BALB/c) have a functional complement system, albeit at lower levels as compared to rats and humans (Ong and Mattes, 1989; Ménoret et al., 2020). Furthermore, compared to mice, rats allow us to draw larger volumes of blood more often and are more convenient to implant human tissues and organoids in anatomically appropriate sites (Adigbli et al., 2020).

Moreover, while NOD-derived immunodeficient mice are restricted by reduced lifespans from thymomas and constraints tied to the *Pkrdc* gene mutation on radiotherapy protocols (Shultz et al., 2007), a problem not observed using other immunodeficient strains of mice without thymomas or carrying *Rag* mutations, immunodeficient rats that do not have *Pkrdc* gene mutations are free from these limitations (Adigbli et al., 2020; Ménoret et al., 2020). Moreover, NOD-derived mice are restricted by reduced lifespans because of thymomas, and immunodeficient mice with *Pkrdc* gene mutations have constraints on radiotherapy protocols (Shultz et al., 2007). These problems are not observed using other immunodeficient strains of mice without thymomas or carrying *Il2rg* or *Rag* mutations. Immunodeficient rats that do not have *Pkrdc* gene mutations are free from these limitations (Adigbli et al., 2020; Ménoret et al., 2020).

Another drawback of HIS mice is that up to 5 months after humanization, there is a large predominance of B over T cells in the blood (Adigbli et al., 2020; Shultz et al., 2007; Agarwal et al., 2020), although at later time points, the percentages of T cells increase and equal those of B cells (Lang et al., 2013).

A key determinant in the generation of HIS mice is the presence of compatible SIRPα on mouse host macrophages, which, when interacting with human CD47, relays “don’t eat me” signals (Adigbli et al., 2020). Since mouse and rat SIRPα are not compatible with human CD47, both short-term humanizations using peripheral blood mononuclear cells (PBMCs) and long-term humanizations with human CD34^+^ cells in both species exhibit low efficacy, even when other human tissues graft successfully (Menoret et al., 2020; Adigbli et al., 2020; Traggiai et al., 2004). NOD mice have a spontaneous mutation in the SIRPa gene that allows interaction with human CD47, and other strains of mice such as BALB/c have been engineered with BACs containing the full human SIRPa sequence (Strowig et al., 2011). This last model does not have any of the drawbacks of the NOD-derived immunodeficient models. Indeed, earlier rat immunodeficient models lacking human SIRPα expression did not show significant immune humanization using either human PBMCs or CD34^+^ cells despite accepting other human tissues (Ménoret et al., 2020; Agarwal et al., 2020; Mashimo et al., 2012). A previous immunodeficient rat model with hSIRPα expression used adult rats and intravenous injection of hCD34^+^ cells resulting in undetectable or very low hCD34-mediated immune humanization in the blood and spleen (Yang et al., 2018). As an additional limitation, these animals showed predominance of B cells and low T cells, as is also the case for HIS mice (Yang et al., 2018).

The rat line used in the present study is *Rag1* and *Il2rg* deficient and expresses high levels of human SIRPα through a human BAC transgene (RRGS) (Ménoret et al., 2020) and thus are similar to NSG mice (*Rag1* and *Il2rg* deficient and with a human compatible SIRPα), a gold standard for immune humanization using CD34^+^ cells. RRGS animals have no detectable T, B, or natural killer (NK) cells and did not have detectable levels of any immunoglobulin but have normal levels of rat granulocytes, monocytes, and macrophages in the spleen (Ménoret et al., 2018). RRGS animals had serum complement levels comparable to those of human serum, whereas mouse NSG and C57BL/6 sera contained low complement activity, as detected in an antibody complement-dependent killing assay (Ménoret et al., 2020). Human SIRPα expression was confined to rat macrophages, and human CD47 interacted with human SIRPα expressed by RRGS monocytes (Ménoret et al., 2020). Without hSIRPα, these animals indefinitely accepted human skin, tumors, and hepatocytes grafts, but did not accept human PBMCs (Ménoret et al., 2018). Remarkably, the introduction of a hSIRPα transgene led to effective immune humanization with human PBMCs (Ménoret et al., 2020). However, while this model addresses short-term human immune responses, such as acute graft-versus-host disease (GVHD) and tumor rejection (Ménoret et al., 2020), it falls short for long-term analyses that humanization with CD34^+^ cells offer.

Cytokine release syndrome (CRS), is a frequent and serious adverse effect of different immune therapies, such as following anti-CD3 muromonab OKT3 monoclonal antibody treatment (Yan et al., 2019). Since CRS needs to be investigated before clinical trials using new immunotherapies, CRS has been modelized in HIS mice, but human cytokines were not observed in NSG mice humanized with hCD34^+^ cells and injected with OKT3 (Matas-Céspedes et al., 2020), unless using also fetal liver and thymus (Yan et al., 2019) or using immunodeficient mice with human hSIRPα and also deficient in *Flk2* (BRGSF) (Martin et al., 2024).

In this paper, we detail a robust protocol for achieving long-term immune humanization in RRGS rats using human CD34^+^ cells. Our approach utilizes irradiated newborn rats and intrahepatic injection of hCD34^+^ cells. The immune humanization rates in HIS rats were comparable to those in BRGSF HIS mice but featured an increased proportion and number of T cells vs. B cells. HIS rats injected with OKT3 showed the depletion of human T cells and the presence of several human cytokines in sera. This model therefore has the potential to be a useful tool for translational immunological studies.

## Results and discussion

Previous results in mice showed that immune humanization was more effective in newborns than in adult animals (Gimeno et al., 2004), and it was later demonstrated that intrahepatic injection after irradiation in newborn mice further increased immune humanization (Traggiai et al., 2004). The protocol of humanization that was used to generate HIS rats took into consideration these previous results in HIS mice.

**Figure 1:**
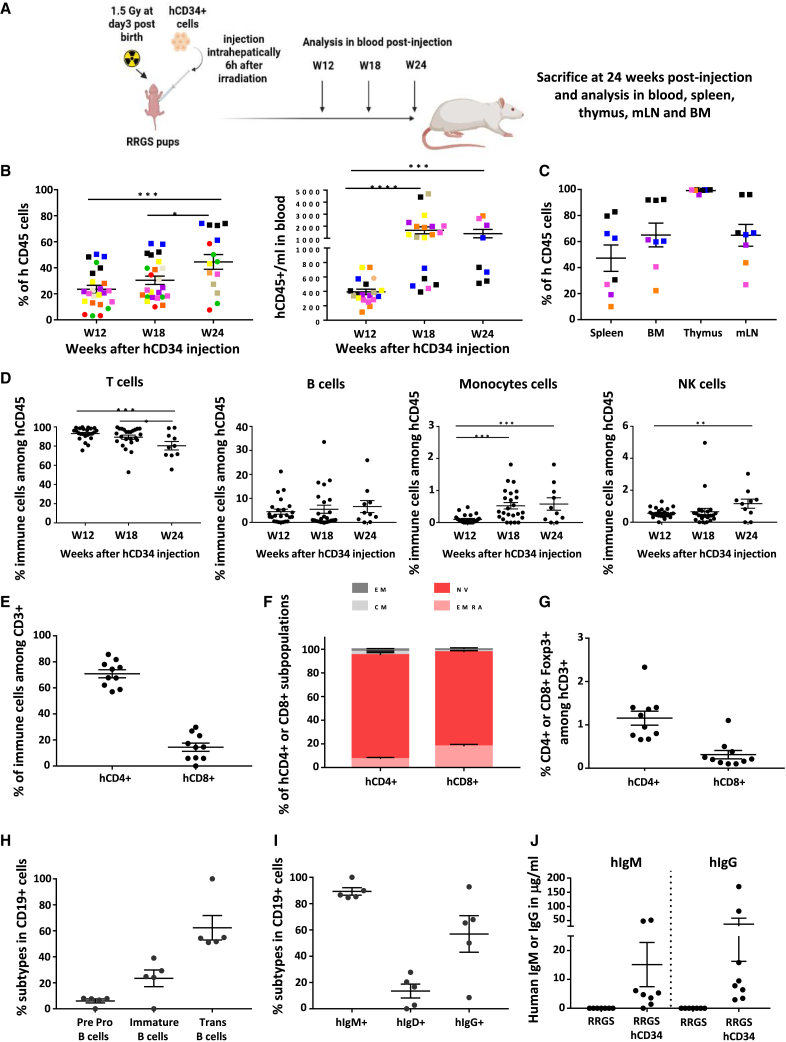
Immune humanization in peripheral blood (A) RRGS newborn animals were sublethaly irradiated and 24 h later injected intrahepatically with human CD34^+^ cells and analyzed for human immune cells in the blood at weeks 12, 18, and 24. At 24 weeks, animals were sacrificed, and their lymphoid organs (spleen, bone marrow [BM], thymus, and mesenteric lymph nodes [mLN]) were also analyzed for human immune cells. (B) Percentages (left, *n* = 8–24) and total numbers (right, *n* = 8–24) of human CD45^+^ cells among PBMCs at different time points. hCD34^+^ cells were from 4 or 9 different donors. Colors in symbols indicate recipients receiving hCD34^+^ cells from different donors. (C) Percentages of hCD45^+^ cells among mononuclear cells in spleen, BM, thymus, and mLN at 24 weeks after hCD34 infusion (*n* = 9). Colors in symbols indicate recipients receiving hCD34^+^ cells from different donors. (D) Percentages in blood of human T (CD3^+^), B (CD19^+^), monocytes (CD14^+^), and NK (CD56^+^) cells among hCD45^+^ cells at 12, 18, and 24 weeks (*n* = 10–24). (E) Percentages in blood of T CD4^+^ or CD8^+^ among human CD3^+^ cells at 24 weeks after hCD34 infusion (*n* = 10). (F) Percentages in blood of T CD4^+^ (left) and CD8^+^ (right) naive (NV, CD45RA^+^CCR7^+^), central memory (CM, CD45RA^-^CCR7^+^), effector memory (EM, CD45RA^-^CCDR7^-^), and terminal effector memory RA^+^ (TEMRA, CD45RA^+^ CCR7^-^) cells at 24 weeks after hCD34^+^ infusion (*n* = 5). (G) Percentages in blood of T CD4^+^FOXP3^+^ and T CD8^+^FOXP3^+^ cells among T cells at 24 weeks after hCD34^+^ infusion (*n* = 10). (H) Percentages in blood of pre-pro B cells (IgM^-^IgD^-^), immature (IgM^+^IgD^-^), and transitional (IgM^+^IgD^+^) CD19^+^ B cells at 24 weeks (*n* = 5). (I) Percentages in blood of IgM^+^, IgD^+^, and IgG^+^ cells among human CD19^+^ cells at 24 weeks after hCD34^+^ infusion (*n* = 5). (J) Levels of human IgM and IgG in serum at 24 weeks (*n* = 8). Each value corresponds to an individual animal and the mean ± SEM is depicted. ^∗^*p* < 0.05, ^∗∗^*p* < 0.005, and ^∗∗∗^*p* < 0.0002.

RRGS newborns were irradiated and injected intrahepatically with hCD34^+^ cells (Figure 1A). At 12 weeks after hCD34 injection, all HIS recipients injected with hCD34^+^ cells showed >3% hCD45^+^ cells among PBMCs (23.6% ± 2.9%). The proportion of hCD45 cells in blood increased at 18 weeks (30.4% ± 3.2%) and at 24 weeks became significantly higher (44.6% ± 5.7%) compared with both 12- and 18-week results (Figure 1B left; Figure S1A). The absolute numbers of hCD45^+^ cells at 18 and 24 weeks (1,660 ± 287/μL, *n* = 19 and 1,383 ± 347/μL, *n* = 8, respectively) increased significantly compared with 12 weeks (382.1 ± 51/μL, *n* = 19) (Figure 1B right).

At 24 weeks, the percentages of hCD45^+^ cells in the spleen (47.2% ± 10.1%) were similar to those in the blood (44.6% ± 5.7%) and even higher in bone marrow (65% ± 9.1%), thymus (99.2% ± 0.5%), and mesenteric lymph nodes (64.8% ± 8.3%) (Figure 1C).

Subset analysis in PBMCs at 12, 18, and 24 weeks showed a majority of T cells (52%–98%) with a slight but significant decrease at 24 weeks (hCD3^+^, 80.4% ± 4.4%). B cells were stable at lower percentages (up to 25%). Monocytes showed a statistically significant increase with time (up to 1.8%). NK cells also showed a statistically significant increase with time (up to 1.9%) (Figures 1D and S1A).

At 24 weeks after humanization, among PBMCs, T CD4^+^ and CD8^+^ cells represented 70.9% ± 3.1% and 14.4% ± 3.2%, respectively (Figures 1E and S1B). In both CD4^+^ and CD8^+^ T cell subsets, naive, central memory, effector memory, and terminal effector memory CD45RA^+^ cells were detected in the blood (Figures 1F and S1C). Among PBMC T cells, FOXP3^+^CD4^+^ and FOXP3^+^CD8^+^ cells were present at 1.15% ± 0.2% and 0.3% ± 0.1%, respectively (Figures 1G and S1B). αβ T and γδ T cells were both present at 98.7% ± 0.3% and 1.3% ± 0.3%, respectively (data not shown). The degree of humanization in the blood of male or female RRGS recipients was not different (Figure S1F).

HIS rats exhibited at 12 weeks in blood equivalent absolute numbers of human CD45^+^ cells (382.1 ± 51/μL, *n* = 19) when compared to HIS BRGSF mice (393 ± 38/μL, *n* = 24) generated with hCD34+ cells from the same vendor (Figure S2A). Interestingly, the absolute number of recipient innate mononuclear cells (mainly monocytes) in HIS RRGS rats is significantly higher than in HIS BRGSF mice (1,498 ± 120.5/μL *n* = 19 vs. 497.8 ± 57.3/μL *n* = 24; *p* < 0.0001) (Figure S2A). This difference in the number of inmonocytes in blood explains why the percentage of hCD45^+^ cells in HIS BRGSF mice is higher than in HIS RRGS rats (Figure S2B). At 12 weeks after humanization, HIS rats and HIS mice showed a very different lymphoid cell subsets distribution in the blood. HIS rats vs. HIS mice showed a much higher absolute number of T cells (367.2 ± 35.6/μL vs. 48.3 ± 12.2/μL), less numbers of B cells (18.1 ± 5.35/μL vs. 275.2 ± 35.6/μL), and comparable numbers of NK cells (2.2 ± 0.3/μL vs. 5.1 ± 0.8/μL) (Figure S2C). At 24 weeks after humanization in HIS rats, the total numbers of hCD45^+^ cells in the spleen were 9.2 ± 2.5 × 10^6^; in the bone marrow, 4.8 ± 1.4 × 10^6^; in the thymus, 4.1 ± 1 × 10^6^; and in the mesenteric lymph nodes, 5 ± 2.1 × 10^4^ (Figure S2D).

At 24 weeks after humanization, among PBMCs and CD19^+^ B cells, there were pre-pro IgM^-^IgD^-^, immature IgM^+^IgD^-^, and transitional IgM^+^IgD^+^ cells (Figures 1H, S1D, and S1E). Among CD19^+^ B cells, 89.3% ± 2.8% were IgM^+^, 13.5% ± 5.3% were IgD^+^, and 56.9% ± 13.9% were IgG^+^ cells (Figures 1I, S1D, and S1E). This B cell subset cell distribution in the blood was similar at 12 and 18 weeks (data not shown). IgM and IgG levels in serum at 24 weeks were of 15.12 ± 7.6 and 37.4 ± 21.2 μg/mL, respectively (Figure 1J), values comparable with what has been observed in immune humanized mice (Dagur et al., 2019), but lower than in human serum (∼1 and 10 mg/mL for IgM and IgG, respectively).

At 24 weeks, in the spleen, most hCD45^+^ cells were T cells, followed by B cells, NK cells, and monocytes (Figure S3A). A similar cell subset distribution to the one in the blood was observed for T (Figure S3B) and B cells with a small percentage of follicular B cells (Figure S3C).

**Figure 2:**
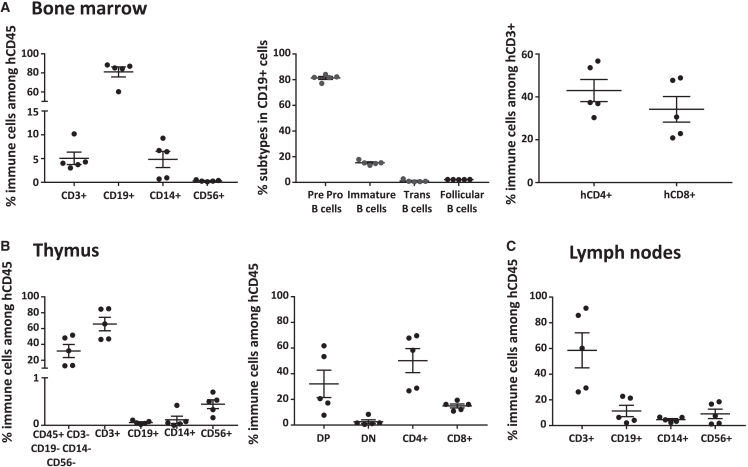
Immune cell subsets in the bone marrow, thymus, and mesenteric lymph nodes RRGS recipients (*n* = 5) were sacrificed 24 weeks after hCD34^+^ cells injection, and mononuclear hCD45^+^ cells of lymphoid organs were analyzed for cell subsets distribution. (A, left) Bone marrow cellular distribution for T (CD3^+^), B (CD19^+^), monocytes (CD14^+^), and NK (CD56^+^) cells; (middle) B cell subsets; and (right) CD4^+^ and CD8^+^ cells. (B, left) In thymus, hCD45^+^CD3^−^CD19^−^CD14^−^CD56^−^, T, B, monocytes, and NK cells; (right) among hCD45^+^CD19^−^CD14^−^CD56^−^, percentages of double CD4^−^ and CD8^−^ cells (DN), double CD4^+^ and CD8^+^ (DP) cells, single CD4^+^ and CD8^+^ cells. (C) In mesenteric lymph nodes, cellular distribution for T (CD3^+^), B (CD19^+^), monocytes (CD14^+^), and NK (CD56^+^) cells.

In the bone marrow, we observed the presence of human CD34^+^ cells (Figure S3D and S3E), T and B cells, monocytes, and NK cells, particularly as expected pre-pro CD19^+^IgM^-^IgD^-^ B cells (Figure 2A left and central). The percentages of CD4^+^ and CD8^+^ T cells were similar (Figure 2A right). We did not observe the development of human CD15^+^CD16^+^ neutrophils neither in the bone marrow (Figure S3D) nor in the blood (data not shown). In the thymus, ∼70% of hCD45^+^ cells were CD3^+^ T cells, whereas 30% were CD3^−^ T cell precursors (hCD45^+^, hCD19^-^hCD56^-^hCD14^-^). Small percentages (<1%) of hCD56^+^ NK cells and very low percentages of hCD19^+^ and hCD14^+^ cells were also detected in the thymus (Figure 2B left). Most T cells were CD4^+^ followed by CD8^+^, CD4^+^/CD8^+^, and CD4^−^/CD8^−^ (Figure 2B right). Human double-negative CD4^−^CD8^−^, double-positive CD4^+^CD8^+^, and single CD4^+^ and CD8^+^ thymocytes were observed in percentages like the ones described in human thymus or in the thymus of CD34^+^ immune humanized mice (Brehm et al., 2010). In the mesenteric lymph nodes, the only lymph nodes observable, most cells were T cells, followed by B cells, NK cells, and monocytes (Figure 2C). We did not observe thymi or mesenteric lymph nodes in RRGS recipients not injected with hCD34^+^ cells (data not shown).

**Figure 3:**
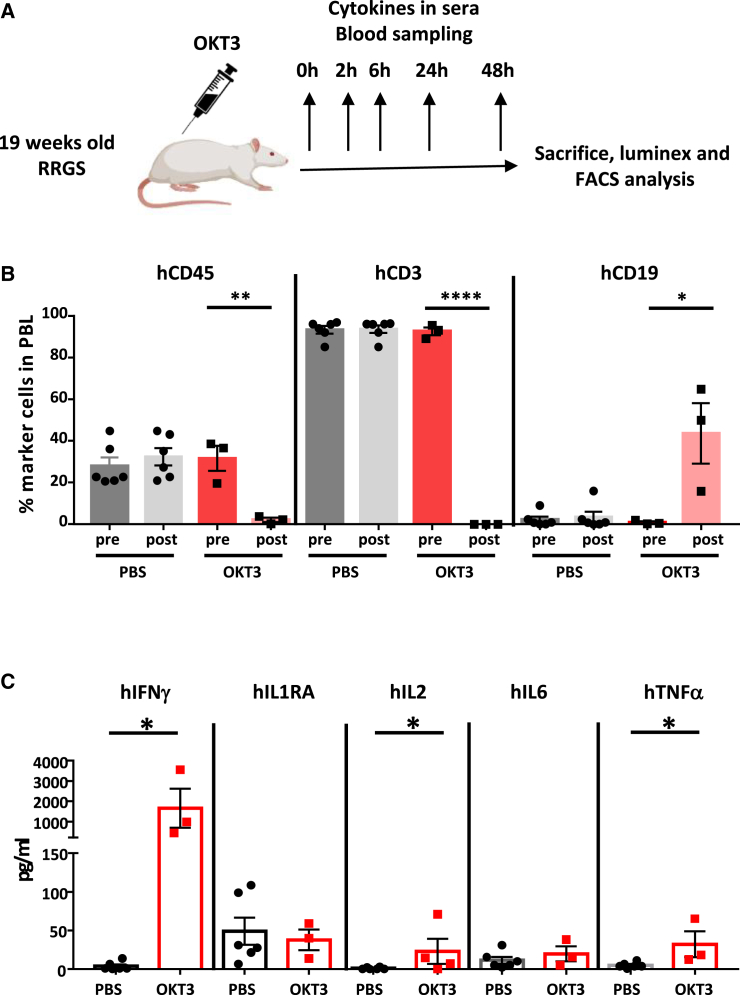
Analysis of T cell depletion and CRS *in vivo* (A) HIS RRGS recipients were injected intraperitoneally with OKT3 or PBS, and serum was harvested at the indicated time points. At 48 h, animals were sacrificed, and human lymphoid populations of peripheral blood were analyzed by cytofluorimetry. (B) Proportion of hCD45, TCR+ T cells, and CD19^+^ cells in HIS rat recipients treated with OKT3 or PBS. (C) Human cytokines were analyzed by Luminex in the sera of HIS rat recipients treated with OKT3 or PBS. OKT3 group, *n* = 3; PBS group, *n* = 6; ^∗^*p* < 0.05, ^∗∗^*p* < 0.005, and ^∗∗∗∗^*p* < 0.0001.

T cell function and the effect of a depleting anti-human antibody were analyzed in HIS rats by injecting the anti-human CD3 antibody muromonab (OKT3) (Figure 3A). At sacrifice at 48 h after injection of OKT3, almost complete depletion of human T cells was observed in the peripheral blood of HIS RRGS recipients, with a parallel increase in the proportion of B cells due to the depletion of T cells (Figure 3B). Along with this, we detected human IFNγ, IL-2, and TNF-α in serum in significative concentrations at 48 h (Figure 3C), with a non-significative increase at 24 h and no detection at 2 h and 6 h (data not shown). Human IL1ra and IL-6 were not increased at any time point (Figure 3C and data not shown).

Thus, following anti-CD3 treatment, human T cells developed in HIS RRGS recipients were activated producing cytokines and were then depleted, similarly to what is observed in humans. Thus, the HIS RRGS model represents a useful model for the analysis of antibodies depleting human lymphoid cells and for potentially detecting dangerous CRS.

RRGS recipients humanized with hCD34 cells never developed acute or chronic GVHD even upon prolonged analysis (40 weeks), whereas NSG mice infused exclusively with hCD34 cells have shown these pathological changes in a fraction of the animals at early (8 weeks) and later time points (12 weeks) with a tendency to increase with time (Sonntag et al., 2015; Fujii et al., 2015; Chandra et al., 2019).

In conclusion, HIS rats exhibit a distribution of lymphoid cell subsets in the blood that mirrors that of human blood. In humans, the proportion of lymphoid cell subsets among lymphocytes (thus excluding granulocytes) also shows a predominance of T (∼70%) over B (∼20%) and NK (∼10%) cells (Apoil et al., 2017; Kokuina et al., 2019). This contrasts with HIS mice, which demonstrate among lymphocytes at 12 weeks a significant B cell predominance (∼80%) over T cells (∼10%) (Adigbli et al., 2020; Shultz et al., 2007; Agarwal et al., 2020), although after >5–6 months following humanization the percentages of T cells increase and equal those of B cells in blood (Lang et al., 2013). The mechanisms that explain the differences of hCD34^+^ differentiation in T and B cells in HIS rats and HIS mice will demand further experimentation. Additionally, although HIS rats and HIS mice have comparable amounts of hCD45 cells in the blood, rats allow for more frequent and larger-volume blood draws (15 mL total blood for a 250 g rat vs. 1.5 mL for a 25 g mice), resulting in higher yields of human immune cells. The absolute numbers of hCD45 cells in lymphoid organs and bone marrow in HIS rats were higher to values in the literature for HIS BRGSF or NSG mice in the spleen (2–5 × 10^6^) and thymus (2–4 × 10^6^), comparable in the bone marrow (5–10 × 10^6^), and lower in mesenteric lymph nodes (1–2 × 10^6^) (Ishikawa et al., 2005; Khosravi-Maharlooei et al., 2020; Yu et al., 2017; Masse-Ranson et al., 2019).

HIS RRGS rats do have limitations, such as a higher cost of breeding per animal due to larger space they occupy in animal facilities compared to mice. The generation of HIS rats demands a higher number of hCD34^+^ cells compared to HIS mice. Some advanced HIS mouse models, such as those using MISTRG (Rongvaux et al., 2014) and BRGSF (*Flk2* deficient) mice, have more myeloid and dendritic cell development (Lopez-Lastra et al., 2017). In this regard, we have generated *Flk2*-deficient rats that in the RRGS background future experiments may show an increase and more diverse immune humanization. Thus, HIS mice are and will continue to be very useful models. HIS rats are not deemed to replace them but now researchers can adapt their experiments to HIS rats if any of their characteristics bring an advantage to their experimental conditions.

Considering the rat’s immune system characteristics, such as their normal complement levels and intact APC function, coupled with their analogous human lymphoid system development post-CD34 transplantation, HIS rats offer a promising useful model for both the analysis and manipulation of human immune responses *in vivo*.

## Experimental procedures

### Resource availability

#### Lead contact

Ignacio Anegon. ianegon@nantes.inserm.fr.

#### Materials availability

RRGS animals will be made available on request, but we may require a payment and/or a completed materials transfer agreement if there is potential for commercial application.

#### Data and code availability

All data will be shared by the lead contact upon request after publication.

### Animals

RRGS animals were generated by crossing *Rag1* and *Il2rg* (Rat Rag1 deficient, Il2rg deficient) (RRG) animals (Ménoret et al., 2018) with a transgenic rat line expressing human SIRPα in rat macrophages (Jung et al., 2016; Ménoret et al., 2020) and maintained under specific pathogen-free conditions. All animal care and procedures performed in this study were approved by the Animal Experimentation Ethics Committee of the Pays de la Loire region, France, in accordance with the guidelines from the French National Research Council for the Care and Use of Laboratory Animals (permit number: Apafis 17618). All efforts were made to minimize suffering. BRGSF (BALB/c *Rag2*^*tm1Fwa*^*IL-2Rγ*_*c*_^*tm1Cgn*^*SIRPα*^*NOD*^*Flk2*^*tm1Irl*^) mice were maintained under specific pathogen-free conditions.

### Immune humanization protocol

Newborn male and female (day 3) RRGS and female BRGSF (day 5) recipients were irradiated (2 and 2.8 Gy, X-ray, respectively), and after 3–6 h, human hematopoietic progenitor CD34^+^ cord blood cells (immunomagnetically purified with anti-CD34 antibodies and purity >95%) (Abcell-bio, Evry-Courcouronnes, France) were defrosted in preheated SCGM medium (CellGenix, Aubagne, France) and DNase I. Immediately after, RRGS recipients received 4.10^5^ hCD34^+^ cells and BRGSF recipients recieved 10^5^ hCD34^+^ cells injected intrahepatically in 30 μL of SCGM medium. hCD34^+^ cells from 15 donors were used to humanize 24 RRGS and 24 for BRGSF described in this study. All animals survived and reconstituted a human immune system. Red blood cells-lysed blood from animals was analyzed at week 12, 18, and 24. Animals were euthanized at week 24, and lymphoid organs were harvested for analysis. The degree of PBMC humanization was calculated as the percentage of human CD45^+^ cells among PBMCs (defined by SSC and FSC parameters)/human CD45^+^ plus rat CD45^+^ cells × 100 or human CD45^+^/human CD45^+^ plus mouse CD45^+^ cells × 100, for HIS rats or HIS mice, respectively (Verma and Wesa, 2020). To calculate the absolute numbers of hCD45, rCD45, or mCD45, we used 123count eBeads Counting Beads (Thermo Fisher Scientific, Illkirch Cedex, France).

Antibodies and cytofluorimetric analyses and ELISAs for anti-human IgM and anti-human IgG were previously described (Ménoret et al., 2010) and are described in detail in the supplementary information section.

### Statistical analysis

Results are presented as means ± SEM. Statistical analysis between samples was performed by a Mann-Whitney test using GraphPad Prism 4 software (GraphPad Software, San Diego, CA, USA).

## Supplemental information

Document S1. Figures S1–S3 and Table S1

Document S2. Article plus supplemental information

## Footnotes

Supplemental information can be found online at https://doi.org/10.1016/j.stemcr.2024.07.005.
