# Supplementary material for: Efficient generation of human immune system rats using human CD34+ cells
Source: Stem Cell Reports. 2024 Aug 15;19(9):1255–63. doi: 10.1016/j.stemcr.2024.07.005 (PMC11411320; doi:10.1016/j.stemcr.2024.07.005)
Supplement: Document S1. Figures S1–S3 and Table S1 [file mmc1.pdf]

Stem Cell Reports, Volume *19*

Supplemental Information

Efficient generation of human immune system rats using human $CD34^+$ cells

Séverine Ménoret, Florence Renart-Depontieu, Gaelle Martin, Kader Thiam, and Ignacio Anegon

Supplemental information

Efficient generation of human immune system rats using human CD34+ cells.

Séverine Ménoret[1,2], Florence Renart-Depontieu[3], Kader Thiam[3] and Ignacio Anegon[2].

Suppl. figure 1.

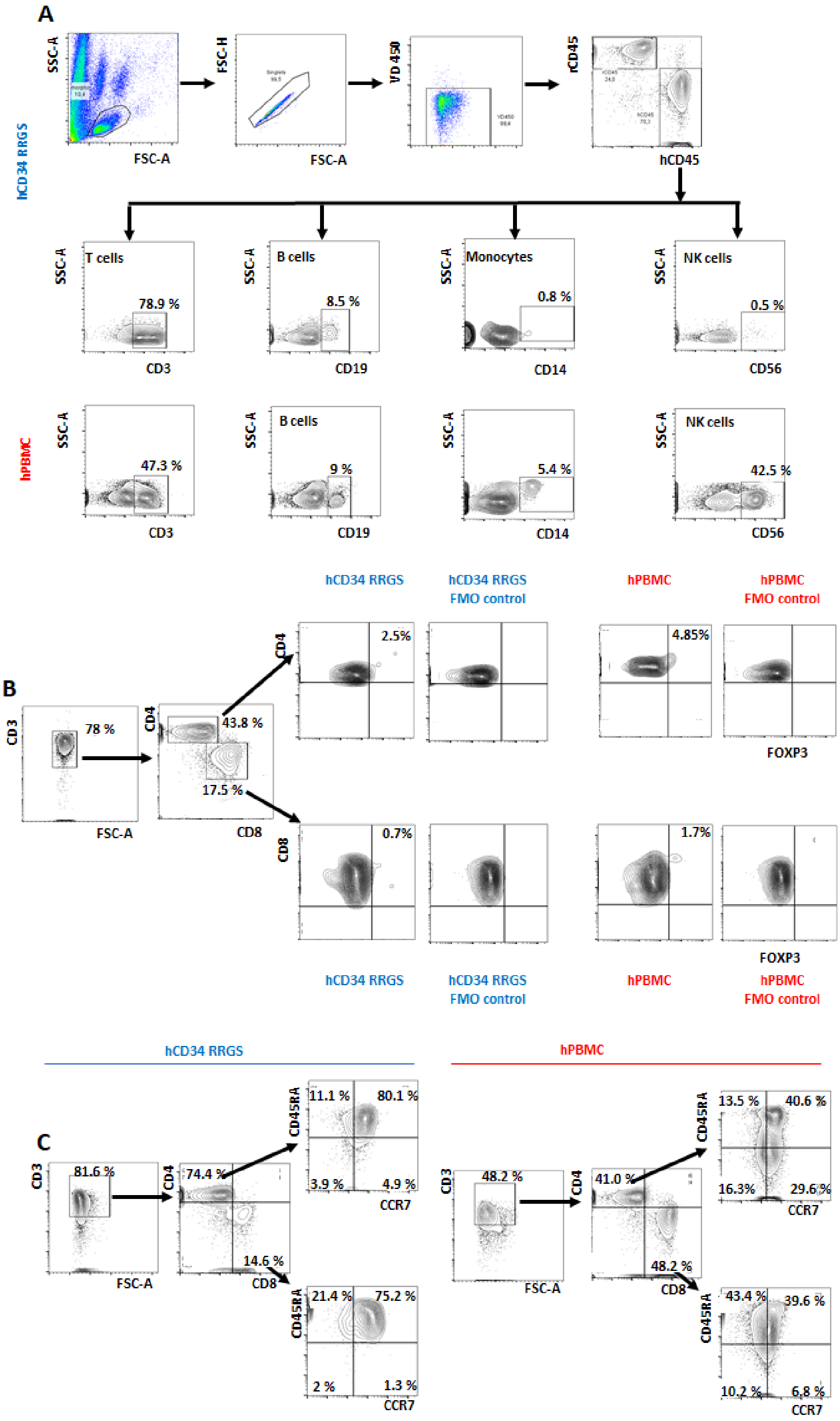

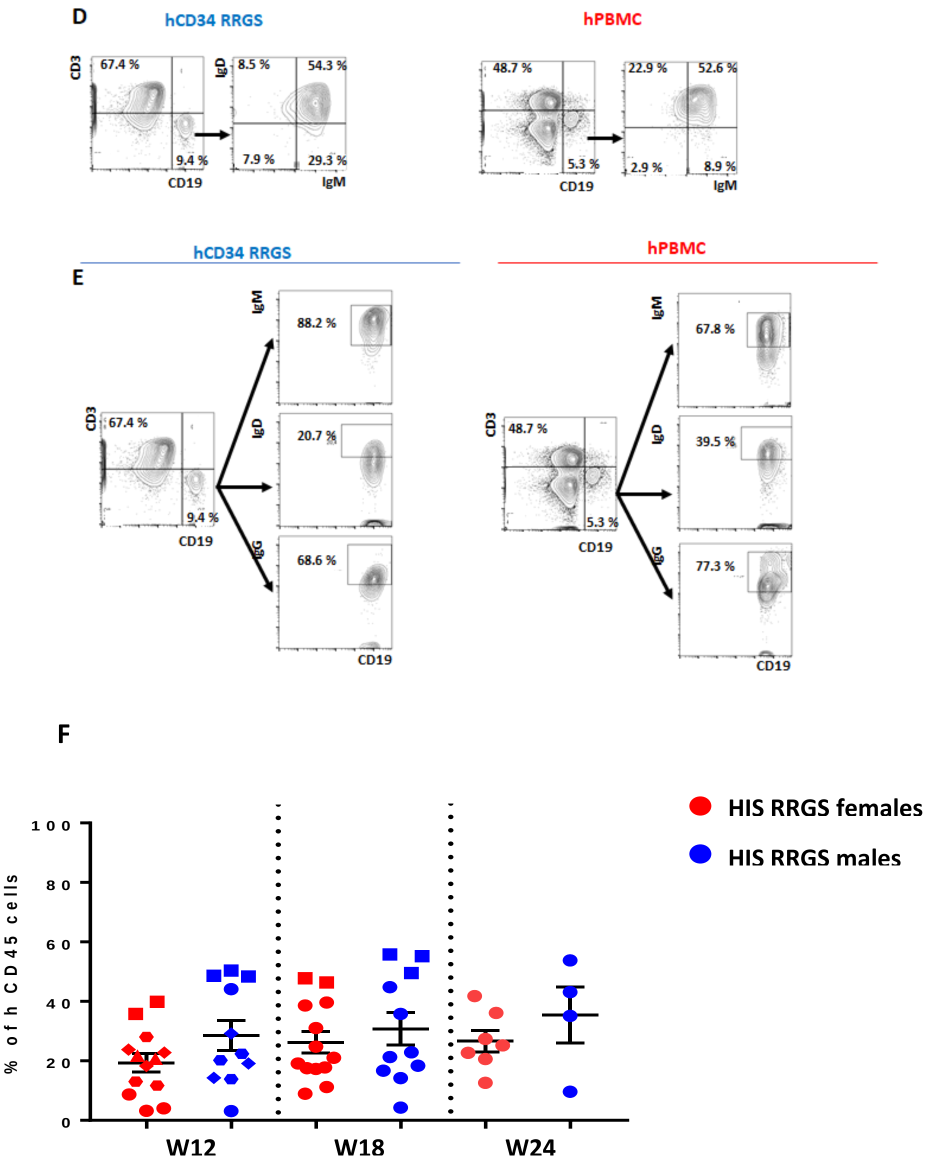

Supplementary figure 1. Gating strategy for human leukocyte subsets analysis and Immune humanization in male and female recipients. Gating strategy for flow cytometry analysis of human blood cells of HIS RRGS recipients. (**A**) Mononuclear cells with lymphocyte characteristics (FCS-A-SSC-A) were defined as single cells (FSC-A FSC-H), viable (VD450 negative) leukocytes (hCD45+, human) were analyzed free from rat CD45+ cells (rCD45 negative) cells. Among hCD45+ were analyzed anti-human antibodies: T cells (SSC-A-CD3+), B cells (SSC-A CD19+), monocytes (SSC-A CD14+) and NK cells (SSC-A CD56+). (**B**) Phenotyping of CD4 or CD8 regulatory T cells: from human T cells (FSC-A CD3+) and then CD4+ or CD8+ cells, the expression of FOXP3. (**C**) Gating strategy for analyzing CD4, CD8 T cells and corresponding subsets, naïve (NV, CD45RA+CCR7+), central memory (CM, CD45RA-CCR7+), effector memory (EM, CD45RA-CCDR7-) and terminal effector CD45RA+ (EMRA, CD45RA+CCR7-) cells. (**D**) Analysis of B cell subpopulations: pre-pro B cells (IgM-IgD-), immature (IgM+IgD-) and transitional (IgM+IgD+) and follicular (IgM-IgD+) CD19+ B cells. (**E**) On human CD45+ cells, B cells (CD19+) were analyzed for expression of B cells markers IgM, IgD and IgG. **(F)** Immune humanization in male and female recipients. Newborn male (n=5-11) and female (n=5-13) RRGS recipients were intrahepatically injected with hCD34+ cells and PBMCs in blood were analyzed at 12, 18 and 24 weeks for the proportion of human CD45+ cells.

Suppl. Fig. 2

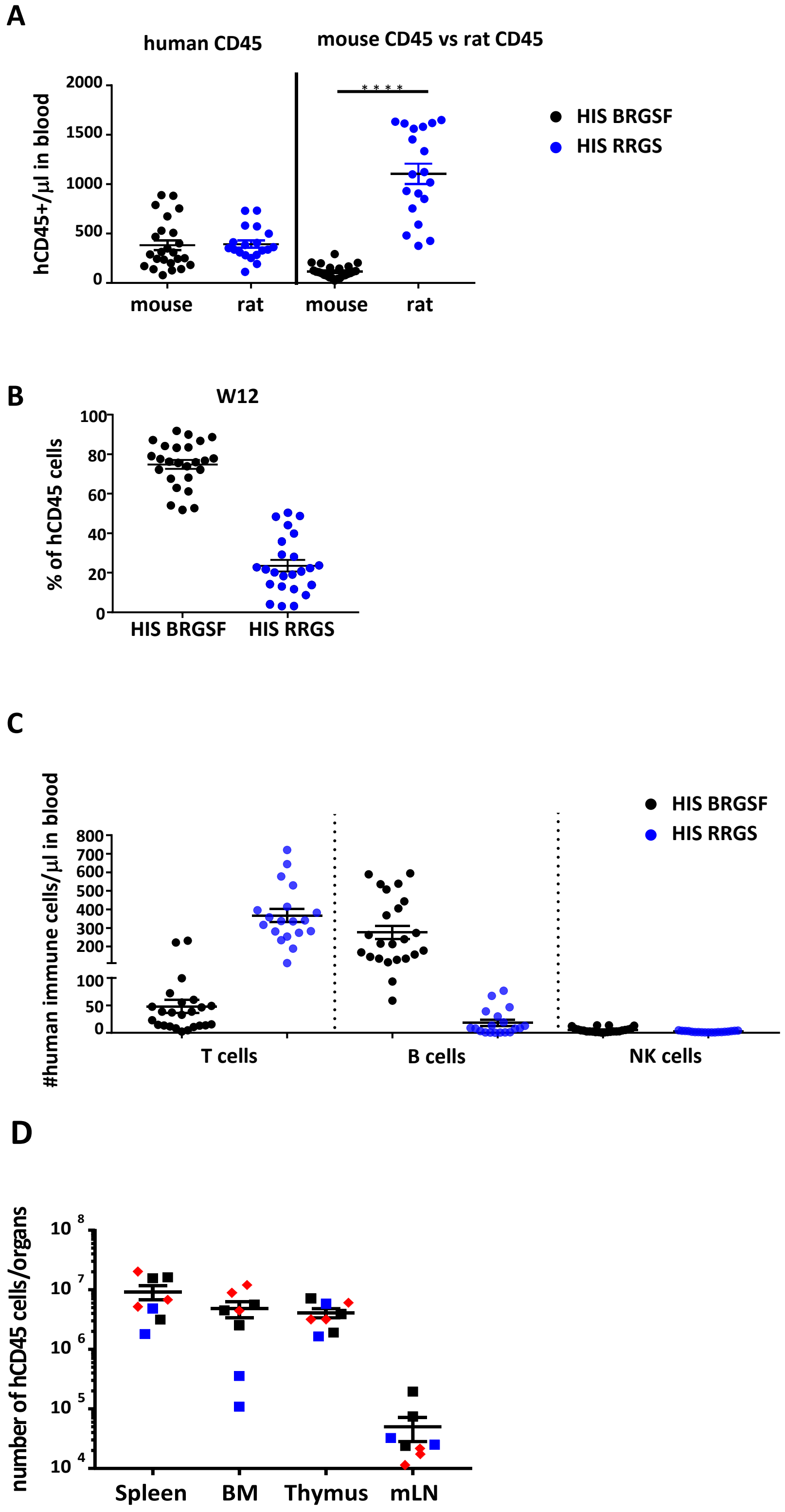

Supplementary figure 2. Analysis of human, rat and mouse lymphoid cell subsets in blood of HIS rats and HIS mice and absolute numbers of hCD45+ cells in lymphoid organs of HIS rats. Blood from HIS BGRSF female mice and HIS RRGS rats was analyzed 12 weeks after intrahepatic transplantation of hCD34+ cells in newborn animals. **(A).** Absolute number of human CD45+ cells in the blood of HIS BGRSF mice (black, n=24, 6 different donors of hCD34+ cells) and of HIS RRGS recipients (blue, n=19, 9

different donors of hCD34+ cells). (**B).** Percentages of hCD45 on HIS BRGF mice and HIS RRGS. (**C)** Absolute numbers of human B, T and NK cells among hCD45+ cells in the blood of HIS BGRSF mice (black) and HIS RRGS rats (blue). **(D)** Absolute numbers of hCD45+ cells in lymphoid organs of HIS rats. HIS RRGS rats were sacrificed at 24 weeks after humanization and the indicated lymphoid organs were processed to analyze the total number of hCD45+ cells per organ. Bone marrow corresponds to one femur. Spleen and thymus correspond to a full organ. Mesenteric lymph nodes (mLN) correspond to 2-3 lymph nodes. Each point represents one animal. HIS rats generated with a given hCD34+ donor are labeled with the same color.

Suppl. Fig. 3

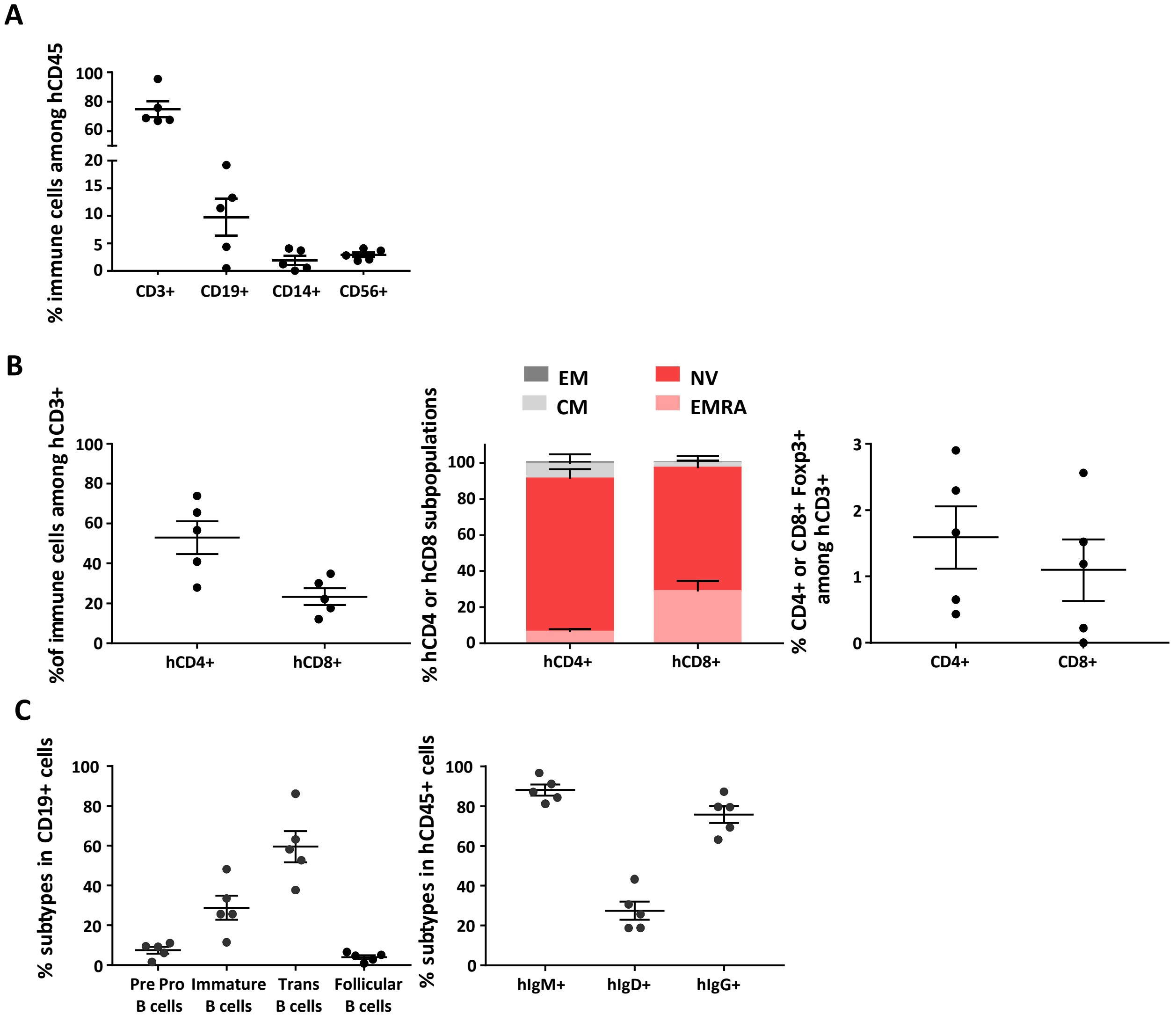
A
% immune cells among hCD45
100
80
60
20
15
10
5
0
CD3+
CD19+
CD14+
CD56+
B
%of immune cells among hCD3+
hCD4+
hCD8+
EM
CM
NV
EMRA
% hCD4 or hCD8 subpopulations
% CD4+ or CD8+ Foxp3+ among hCD3+
CD4+
CD8+
C
% subtypes in CD19+ cells
Pre Pro B cells
Immature B cells
Trans B cells
Follicular B cells
% subtypes in hCD45+ cells
hIgM+
hIgD+
hIgG+

D

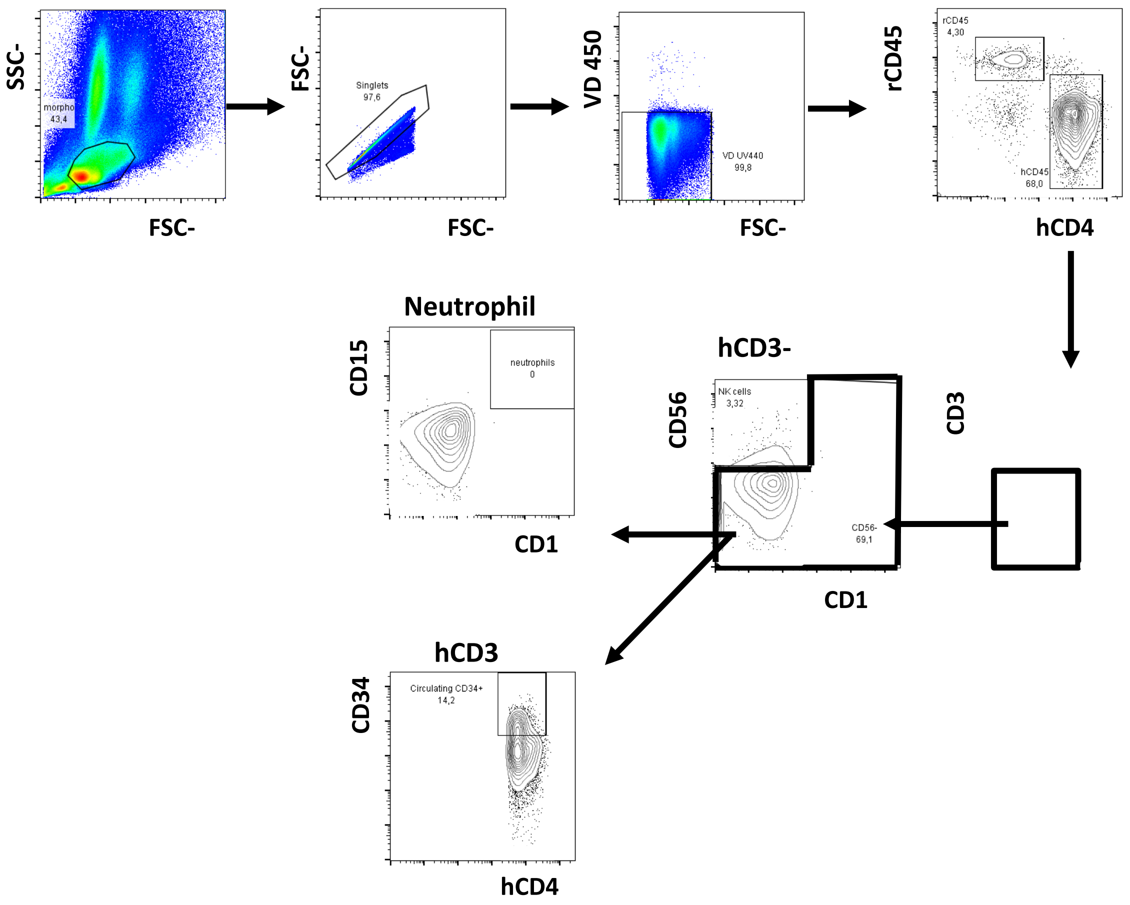

E

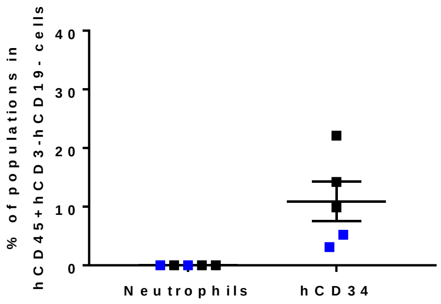

Supplementary figure 3. Human immune cell subsets in the spleen and analysis of human CD34+ and PMN (CD15+) in bone marrow. RRGS recipients (n=5) were sacrificed 24 weeks after hCD34+ injection and analyzed for hCD45+ cell subsets distribution. **A.** Percentages T (CD3+), B (CD19+), monocytes (CD14+ and NK (CD56+) cells. (**B, left)** The ratio of CD4+ and CD8+ cells; **middle,** percentages of naïve, central memory (CM), effector memory (EM) and terminal effector RA+ (TEMRA) cells among CD4+ and CD8+ cells; **right**, percentages of T CD4+ or CD8+ FOXP3+ cells. (**C, left)** Percentages of pre-pro B

cells (IgM-IgD-), immature (IgM+IgD-) and transitional (Trans IgM+IgD+) follicular (IgM-IgD+) CD19+; **right,** Percentages of IgM+, IgD+ and IgG+ cells among human CD19+ cells. **(D)** Analysis of human CD34+ and PMN (CD15+) in bone marrow. HIS RRGS recipients (n=5) were sacrificed 24 weeks after hCD34+ injection and analyzed for hCD34+ and hCD15 cells in the bone marrow. Gating strategy for flow cytometry analysis of human CD34+ and CD15+ cells. **(E)** Percentage of hCD34+ cells among hCD45+ cells in bone marrow. n=5 animals, different colors for 2 different hCD34+ donors.

Supplementary Table 1.

**Target**	**Label**	**Clone**	**Catalog number**	**Origin**
hCCR7 (CD197)	BV510	3D12	583449	BD Biosciences
hCD14	BUV737	M5E2	612763	BD Biosciences
hCD19	FITC	HIB19	555412	BD Biosciences
hCD3	PE	Hit3a	555340	BD Biosciences
hCD4	BV711	RPA-T4	568371	BD Biosciences
hCD45	PerCp/Cy5.5	HI30	564105	BD Biosciences
hCD45RA	BV395	HI100	568712	BD Biosciences
hCD56	BV605	NCAM16.2	562780	BD Biosciences
hCD8	BUV 737	RPA-T8	749367	BD Biosciences
hCD8	BV605	RPA-T8	569310	BD Biosciences
hFOXP3	Percp-Cy5.5	PCH101	45-4776-42	eBioscience
hIgD	BUV 395	IA6-2	563813	BD Biosciences
hIgG	BV 786	G18-145	564230	BD Biosciences
hIgM	BV510	G20-127	563113	BD Biosciences
hTCRab	FITC	IP26	306705	Biolegend
hTCRgd	BV421	B1	562560	BD Biosciences
rCD45biot	biotinylated	OX1+OX30		Homemade
Streptavidin PE-Cy7	PE-Cy7		557598	BD Biosciences

Experimental procedures.

Antibodies and cytofluorimetric analyses. Single-cell suspensions from the PBMCs, spleen, thymus, bone marrow, and mesenteric lymph nodes were prepared as described previously (Ménoret et al., 2010). Cell suspensions were analyzed for human CD45+ cells and human cell subsets as well as for rat CD45+ cells using differently labeled antibodies **(Supplementary table 1).** Cells were incubated with the antibodies for 30 minutes at 4°C, and the analysis was performed with a FACSVerse and Celesta

equipment (BD Biosciences, Le Pont de Claix, France) and FlowJo software. The limit of 0.1% of positive cells was considered as a threshold for humanization of different cell populations, as it has been used in the past with HIS mice (Dick et al.,1991; Piau et al., 2023).

ELISA anti-human IgM and anti-human IgG. Levels of human IgM and IgG were determined by ELISA. Multisorp plates (NUNC, Thermo Scientific™, Saint-Herblain, France) were coated overnight at 4°C with anti-human IgM or IgG capture antibody (STEMCELL Technologies SARL, Saint Egrève, France). After washing and blocking the wells, diluted samples were applied for 2 hours at room temperature. Human serum with known concentrations of IgM and IgG (Stem Cell) was used as a standard. Secondary anti-human IgM or IgG alkaline phosphatase conjugated antibody were added. ELISAs were developed using p-nitrophenyl phosphate, and the reaction was stopped using stop solution. Plates were analyzed using a Tecan plate reader at 405nm.

Ménoret S, Iscache AL, Tesson L, Rémy S, Usal C, Osborn MJ, Cost GJ, Brüggemann M, Buelow R, and Anegon I. (2010). Characterization of immunoglobulin heavy chain knockout rats. Eur J Immunol. 40, 2932-41

Dick, J.E., Pflumio, F. and Lapidot, T. (1991). Mouse models for human hematopoiesis. Semin Immunol 3, 367-78.

Piau, O., Brunet-Manquat, M., L'Homme, B., Petit, L., Birebent, B., Linard, C., Moeckes, L., Zuliani, T., Lapillonne, H., Benderitter, M. et al. (2023). Generation of transgene-free hematopoietic stem cells from human induced pluripotent stem cells. Cell Stem Cell 30, 1610-1623.e7.
